# 

**Published:** 1876-09

**Authors:** 


### CONTENTS:

ARTICLE I.—Proceedings of the Sixteenth Annual Meeting of the American Dental Association....... 193

ARTICLE II.—American Academy of Dental Science....... 211

ARTICLE III—Methods of, and Materials for, Filling Teeth....... 216

ARTICLE IV.—In what does the Improvement in Dentistry during the last fifteen years consist? By Dr. G. V. Black....... 225

### EDITORIAL, ETC.

The Recent Meeting of the American Dental Association....... 233
The Centennial....... 235
OBITUARY.—Professor Elias Wildman, M. D., D. D. S....... 236

### MONTHLY SUMMARY.

To Cut Cold Steel with Soft Iron....... 237

Carbolic Acid Poisoning....... 238

Fracture of Maxillæ with Profuse Hemorrhage....... 238

Chromic Acid for Warts....... 239

Electro-Magnetism as a Motive Power....... 240

Antiseptic Property of Borax....... 240

